# The complete chloroplast genome sequence of *Anemone reflexa* (Ranunculaceae)

**DOI:** 10.1080/23802359.2020.1860710

**Published:** 2021-02-05

**Authors:** Ningning Zhang, Yang Lu, Zhenqiang Zhang

**Affiliations:** a Academy of Chinese Medical Sciences, Henan University of Chinese Medicine, Zhengzhou, PR China; b School of Agricultural Sciences, Zhengzhou University, Zhengzhou, PR China

**Keywords:** Anemone, Ranunculaceae, chloroplast genome, phylogenetic analysis

## Abstract

The complete chloroplast genome sequence of *Anemone reflexa* exhibited a typical quadripartite structure with two inverted repeats (IRa and IRb) of 31,260 bp, a large single-copy region (LSC) of 80,767 bp, and a small single-copy region (SSC) of 17,623 bp. The chloroplast genome encoded a set of 133 genes, comprised of 89 protein coding genes, 36 tRNA genes and 8 rRNA genes. Phylogenetic analysis suggested that *A. reflexa* was closely related to *A. raddeana*. The complete chloroplast genome sequence of *A. reflexa* will provide valuable genetic resources for molecular identification and phylogenetic analysis of *Anemone*.

The *Anemone* genus, belonging to the family Ranunculaceae, has long been used in folk medicine and worldwide ethnomedicine, and more than 50 species in this genus have ethnopharmacological uses (Hao et al. 2017). *Anemone reflexa* is distributed in China, Korea, Mongolia and Siberia, and usually grows in open forests and valleys (Wang and Ziman 2001). Due to morphological similarity, rhizomes of *A. reflexa* are usually collected together with other traditional herbal medicines such as that of *A. altaica* and *A. raddeana* in the local market. The whole plastomes can be used as super-barcodes for accurate classification and identification for closely related species (Li et al. 2015; Fu et al. 2019). In the present study, the complete chloroplast genome sequence of *A. reflexa* is reported contributing to the molecular identification and phylogenetic analysis of *Anemone*.

The fresh and healthy leaves of an individual of *A. reflexa* in Longyuwan, Luanchuan county, Henan province (111°47′50.80″E, 33°40′50.68″N, 1733 m) were collected and stored in plastic bags with silica gel until DNA extraction. The specimen was deposited at Zhengzhou University (accession number: LY2019041111). Total genomic DNA was extracted according to a modified CTAB method (Doyle and Doyle 1987) and the shotgun library with an average insert size of 500 bp fragments was constructed. High-throughput DNA sequencing was conducted using the *NovaSeq6000* System with S1 FlowCell (Illumina Inc., San Diego, CA) with 150 bp paired-end reads in Sangon Biotech (Shanghai) Co., Ltd. Approximately 3.3 Gb raw data were generated and used to assemble the chloroplast genome by GetOrganelle (Jin et al. 2020).Genome annotation was performed by GeSeq (Tillich et al. 2017) with *A. raddeana* as the reference and checked manually using Geneious Prime (Kearse et al. 2012). The annotated chloroplast genome of *A. reflexa* was submitted to GenBank with accession number MW043774. The complete chloroplast genome of *A. reflexa* and other eleven species from Ranunculaceae were aligned with MAFFT (Katoh and Standley 2013). Take *Actaea dahurica* as the outgroup, the maximum likelihood (ML) tree was constructed by RAxML (Stamatakis 2014) based on rapid bootstrap algorithm with the GTRGAMMA model and 1000 bootstrap replications.

The complete chloroplast genome of *A. reflexa* is 1,60,910 bp in length, exhibiting a typical quadripartite structure with two inverted repeats (IRa and IRb: 31,260 bp). The large single-copy region (LSC) and the small single-copy region (SSC) separated by IRs are 80,767 bp and 17,623 bp, respectively. Totally, 133 genes were annotated, including 89 protein coding genes, 36 tRNA genes and 8 rRNA genes. The GC content of the whole genome, IRs, LSC and SSC regions are 37.64%, 41.90%, 35.78% and 31.08%, respectively. Phylogenetic analysis results indicate *A. reflexa* is closely related to *A. raddeana* (Figure 1), which is congruent with previous molecular results (Hoot et al. 2012; Zhai et al. 2019).

**Figure 1.**
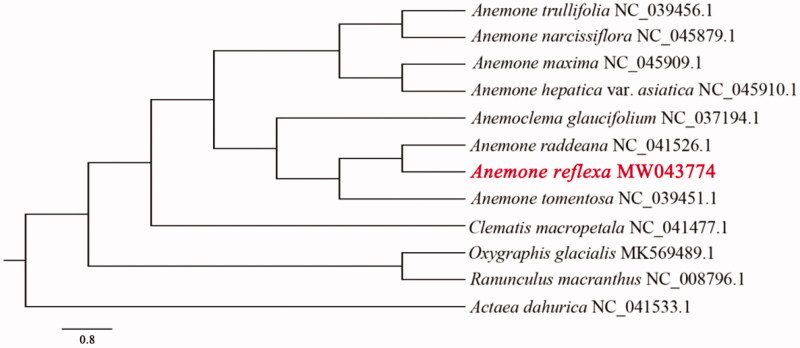
Phylogenetic tree inferred by maximum likelihood (ML) method based on the complete chloroplast genome sequences of 12 species. The numbers on the branches are RAxML bootstrap supports based on 1000 replicates.

## Data Availability

The data that newly obtained at this study are openly available in the NCBI (https://www.ncbi.nlm.nih.gov/) under accession number of MW043774.
